# Small area synthetic estimates of smoking prevalence during pregnancy in England

**DOI:** 10.1186/s12963-015-0067-8

**Published:** 2015-12-09

**Authors:** Lisa Szatkowski, Samantha J. Fahy, Tim Coleman, Joanna Taylor, Liz Twigg, Graham Moon, Jo Leonardi-Bee

**Affiliations:** Division of Epidemiology and Public Health, University of Nottingham, School of Medicine, Clinical Sciences Building, Nottingham City Hospital, Nottingham, NG5 1PB UK; Division of Primary Care, Medical School, Queen’s Medical Centre, University of Nottingham, School of Medicine, Nottingham, NG7 2UH UK; University of Southampton, Geography and the Environment, University Road, Southampton, SO17 1BJ UK; Department of Geography, University of Portsmouth, Buckingham Building, Lion Terrace, Portsmouth, PO1 3HE UK

**Keywords:** Smoking, Pregnancy, Synthetic estimation

## Abstract

**Background:**

Complete and accurate data on maternal smoking prevalence during pregnancy are not available at a local geographical scale in England. We employ a synthetic estimation approach to predict the expected prevalence of smoking during pregnancy and smoking at delivery by Primary Care Trust (PCT).

**Methods:**

Multilevel logistic regression models were used with data from the 2010 Infant Feeding Survey and 2011 Census to predict the probability of mothers (a) smoking at any point during pregnancy and (b) smoking at delivery, according to age, deprivation, and the ethnic profile of the home area. These probabilities were applied to demographic information on mothers giving birth from 2010/11 Hospital Episode Statistics data to produce expected counts, and prevalence figures, of smokers by PCT, with Bayesian 95 % credible intervals. The expected prevalence of smoking at delivery by PCT was compared with midwife-collected Smoking at the Time of Delivery (SATOD) data using a Bland-Altman plot.

**Results:**

The expected prevalence of smoking during pregnancy by PCT ranged from 8.1 % (95 % CI 5.6–1.0) to 31.6 % (27.5–34.8). The expected prevalence of smoking at delivery ranged from 2.5 % (1.4–4.0) to 17.1 % (13.7–20.4). Figures for expected smoking prevalence at delivery showed some agreement with SATOD, though SATOD data were generally higher than the synthetic estimates (mean difference 2.99 %).

**Conclusions:**

It is possible to derive good estimates of expected smoking prevalence during pregnancy for small areas, potentially at much lower cost than conducting large surveys. Such data may be useful to help plan and commission smoking cessation services and monitor their effectiveness.

**Electronic supplementary material:**

The online version of this article (doi:10.1186/s12963-015-0067-8) contains supplementary material, which is available to authorized users.

## Background

Maternal smoking during pregnancy causes significant adverse health outcomes for babies in-utero, during the neonatal period and beyond [1]. However, data available to quantify smoking prevalence during pregnancy in England are of variable extent and quality. Hospital midwives collect data on women’s smoking status at the time of delivery (SATOD [2]), which, in the absence of any other sources of routine data, are currently used as a standard. However, decisions regarding how SATOD data are collected are made locally (and thus may vary geographically), large amounts of data are missing in some areas of the country (up to 10 % for deliveries in one Primary Care Trust in 2012–13 [3]), and questions have been raised about data accuracy [4]. For example, some midwives report finding it difficult to discuss smoking with women at delivery, and some maternity service providers do not prioritize this item of data collection [4].

New information standards have recently been introduced requiring all National Health Service (NHS) maternity services in England to routinely collect self-reported information on smoking status and daily cigarette consumption from pregnant women attending their booking appointment (at approximately 8–12 weeks’ gestation) [5]; however, it will be some time before the quality of these data is known. Nationally, no data on smoking are collected routinely throughout pregnancy. Survey data are available from the Infant Feeding Survey (IFS), which asks women, albeit retrospectively, about smoking at various points in their pregnancy. However, the IFS is only conducted every five years, is based on a small sample of births (a target sample size of 5,000 mothers to complete all three follow-up surveys [6]), and cannot give direct estimates of smoking prevalence for small geographical areas.

The availability of good quality data on smoking prevalence during pregnancy for small geographical areas, such as those based on health service geography, would be useful to help with planning services and monitoring the effectiveness of smoking prevention and cessation interventions [7]. However, data for small areas are difficult to acquire—surveys are time-consuming and costly, likely prohibitively so, as many pregnant women would need to be sampled in each small area in order to give precise estimates of smoking behavior. Previously, synthetic estimation techniques have been used to predict the expected prevalence of various health behaviors and indicators for small geographical areas in England, including general adult smoking prevalence [8], excess alcohol intake [9], obesity [10], and diabetes [11]. These model-based estimates have been shown to be valid [12].

Here we use synthetic estimation to predict maternal smoking prevalence at delivery as well as at any point during pregnancy for small health areas in England, and compare the former to SATOD data.

## Methods

### Stage 1: predicting the probability of maternal smoking in pregnancy and at delivery

We used data from mothers in England who completed Stage 1 (at 4–10 weeks post-delivery) of the 2010 IFS. Using self-reported information on smoking status we created two binary variables to identify whether women smoked at any point during their pregnancy, including between conception and confirmation of pregnancy, and whether they were smoking at delivery (see Additional file 1 for details of the survey questions asked). We identified the age group of each woman (16–19, 20–24, 25–29, 30+ years) and the Index of Multiple Deprivation (IMD) quintile of the Lower Super Output Area (LSOA, of which there are 32,482 in England) of her home address. At the time of the 2010 IFS survey, 151 Primary Care Trusts (PCTs) were responsible for commissioning community, primary, and secondary healthcare services in England, including the provision of National Health Service Stop Smoking Services (NHS SSS) and specialist Stop Smoking Services for Pregnancy (SSSP). IFS respondents’ PCTs of residence, not routinely released with the IFS dataset, were provided by the NHS Health and Social Care Information Centre. Women were dropped from the analysis if one or more of their smoking status, age, IMD quintile, or PCT of residence were missing.

As smoking behaviors are strongly associated with ethnicity [13], we used data from the 2011 Census [14] to derive an area-level variable indicating the percentage of women in each PCT who were not of white or mixed ethnicity. The reason for using this categorization was that examination of IFS data suggested that the prevalence of smoking in pregnancy was similar in women of white and mixed ethnicity, and lower amongst women of Asian or Asian British, Black or Black British, and Chinese or other ethnicities. This variable was centered, meaning that the value for each PCT was subtracted from the mean. An area-level variable was used because individual-level ethnicity was missing for a large number of women in our data (see *Sensitivity analysis).*

With MLwiN version 2.30 [15], run from within Stata [16] using the ‘runmlwin’ command [17], we built two separate multilevel, multivariable logistic regression models to predict the probabilities of a woman reporting smoking at any point during pregnancy and at delivery. Age group and IMD quintile, and the interaction between these, were included as individual-level predictor variables (though, as noted above, IMD quintile is not strictly an individual-level variable as it is based on the LSOA of residence) and the percentage of women not of white or mixed ethnicity as an area-level predictor. Centering of the ethnicity variable means that the intercept term is interpreted as the expected smoking prevalence when the proportion of women not of white or mixed ethnicity is at its mean level and age group and IMD are at the baseline category of these variables. PCT was modeled as a random intercept. Parameters were only retained in the model if they were statistically significant (*p <* 0.05). An Iterative Generalized Least Squares (IGLS) model was run initially to provide starting estimates for a final Markov Chain Monte Carlo (MCMC) model [18] with 50,000 burn-ins and one million simulations. The fit of the final MCMC models was assessed by examining the trajectories of the simulations, distribution of model parameter estimates and the Estimated Sample Size (ESS) for each parameter.

### Stage 2: estimating the number of smokers by PCT

Using data from 2010/11 Hospital Episode Statistics (HES) [19] we identified all deliveries in NHS hospitals in England between April 1, 2010 and March 31, 2011. From the HES dataset we also extracted age group (coded as above), IMD quintile, and PCT of residence of the mother, dropping records where one or more of these variables were not recorded (see *Results* for details of data dropped). We linked the area-level ethnicity variable derived from Census data to each mother’s record, matched on her home PCT. These HES data were collapsed within PCTs by age group and IMD quintile to generate a count of the number of deliveries in the study period for each combination of categories of these two variables.

The parameter estimates from the MCMC models of IFS data were used to calculate the probability of a woman smoking according to her age group and IMD quintile. For each PCT, these probabilities were multiplied by the number of deliveries recorded in the HES data to women with each combination of age group and IMD, and then weighted for the percentage of women living in her PCT who were not of white or mixed ethnicity, to give an estimate of the number of women per stratum who smoked. The number of deliveries and estimated number of smokers in each stratum were summed by PCT to derive an estimate of smoking prevalence. Using methodology that has previously been adopted elsewhere [20] we calculated a Bayesian 95 % credible interval for smoking prevalence for each PCT.

A flow chart summarizing our methods and providing further statistical details on the derivation of the credible intervals is provided as an additional file (Additional file 1).

### Sensitivity analysis

We re-ran stages one and two of our estimation procedures using ethnicity recorded at an individual-level within the IFS and HES where these data were available, instead of using the area-level ethnicity variable derived from census data. This necessitated dropping the approximately 8 % of deliveries recorded in HES where ethnicity was not documented. While counts of expected numbers of smokers by PCT were likely underestimated, estimates of smoking prevalence were similar to those derived from models using ethnicity as an area-level variable. For brevity we present here only results from models where ethnicity is operationalized as an area-level variable, favoring these as fewer deliveries had to be excluded from the HES data, and thus there was less underestimation of the absolute expected number of smokers.

### Model validation

We used several of the approaches to validation of synthetic estimates which have been employed elsewhere [12]. First, we examined the relative magnitude and direction of the parameter estimates from the MCMC logistic regression models to assess whether these were in line with existing literature. A good synthetic estimation model ought to explain at least 40 % of the variation between areas [21] and so from the IFS models we calculated the percentage of variation in smoking prevalence between PCTs explained by the parameters included in the final, parsimonious models. We also examined normal probability plots of the residuals from the IFS models, and calculated Moran’s I to examine the extent of spatial autocorrelation between model area-level residuals; a value of Moran’s I greater than 0.1 was considered as a sign of model invalidity [12].

We graphically compared our synthetic estimates to estimates of smoking prevalence by PCT derived directly from IFS data (which should generally be unbiased, just underpowered where the IFS surveyed only a few people in an area [12]). We checked for heteroscedasticity by subtracting the synthetic estimates from the IFS estimates for each PCT and plotted these residuals against the synthetic estimates.

Finally, we drew a Bland-Altman plot to compare our synthetic estimates of smoking prevalence at delivery by PCT with SATOD data from 2010/11 [22] to assess the extent of agreement between the two measures.

### Ethical approval

IFS data were obtained from the UK Data Service. No ethics approval was required for their use. Census data are available freely online. Ethics approval for the use of the HES data was obtained from the NHS Health and Social Care Information Centre (reference number NIC-166107-GT9RJ).

## Results

7,336 mothers completed the 2010 IFS stage one survey, though of these 35 (0.5 %) had missing data for age, 10 (0.1 %) were missing IMD, and 189 (2.6 %) did not give information about their smoking during pregnancy. After dropping women with one or more missing variables, data were available from 7,085 (96.6 %) respondents for subsequent modeling. Table 1 shows parameter estimates for the associations between predictor variables and smoking during pregnancy and at delivery from the final MCMC models.

**Table 1:** Parameter estimates from the MCMC models built using IFS data

	Association with smoking ever during pregnancy		Association with smoking at delivery	
	β (SE)	OR (95 % CI)	β (SE)	OR (95 % CI)
Age group (baseline = 30+ years)				
25–29	0.500 (0.075)	1.73 (1.50–2.01)	0.366 (0.104)	1.44 (1.18–1.77)
20–24	1.132 (0.095)	3.10 (2.57–3.74)	0.967 (0.117)	2.63 (2.09–3.31)
Under 20	1.647 (0.176)	5.19 (3.68–7.33)	1.445 (0.194)	4.24 (2.90–6.20)
Quintile of Index of Multiple Deprivation (baseline = 1 most deprived)				
2	−0.254 (0.089)	0.78 (0.65–0.92)	−0.446 (0.110)	0.64 (0.52–0.79)
3	−0.496 (0.096)	0.61 (0.50–0.74)	−0.765 (0.125)	0.47 (0.36–0.59)
4	−0.582 (0.110)	0.56 (0.45–0.69)	−1.076 (0.157)	0.34 (0.25–0.46)
5 (least deprived)	−1.025 (0.123)	0.36 (0.28–0.46)	−1.713 (0.193)	0.18 (0.12–0.26)
Individual-level interactions				
20–24 X IMDQ4	0.652 (0.226)	1.92 (1.23–2.99)	0.848 (0.278)	2.33 (1.35–4.03)
20–24 X IMDQ5	1.017 (0.253)	2.76 (1.68–4.54)	1.078 (0.346)	2.94 (1.49–5.79)
<20 X IMDQ3	1.379 (0.573)	3.97 (1.29–12.2)	1.150 (0.507)	3.16 (1.17–8.53)
<20 X IMDQ4^a^	0.730 (0.461)	2.08 (0.84–5.12)	0.713 (0.499)	2.04 (0.77–5.43)
<20 X IMDQ5	1.577 (0.665)	4.84 (1.31–17.8)	2.589 (0.651)	13.3 (3.72–47.7)
Area-level variables				
% females not of white or mixed ethnicity	−0.019 (0.002)	0.98 (0.98–0.99)	−0.023 (0.003)	0.98 (0.97–0.98)
Constant	−1.519 (0.076)		−2.054 (0.096)	
Level 2 variance	0.012 (0.012)		0.017 (0.020)	

^a^Parameter retained in parsimonious model despite non-significance as the number of women in this strata was very small (*n =* 25), limiting power. All other excluded interaction parameters were highly non-significant

These parameter estimates are broadly as expected and in line with existing literature—the likelihood of smoking increases with decreasing age group and increasing levels of deprivation, though younger mothers are more likely to be smokers regardless of their level of deprivation. As the percentage of women in an area who are not of white or mixed ethnicity increases, the likelihood of smoking falls. The predictor variables included in the two models appear to explain a high percentage of the variation in smoking prevalence between PCTs (86.4 % of variation in smoking at delivery and 83.5 % of variation in smoking during pregnancy). Normal probability plots of PCT-level residuals from the IFS models (see Additional file 2) showed no substantial departures from normality and choropleth maps of the residuals (Additional file 3) suggested there was no spatial clustering (Moran’s I = 0.019 for smoking ever during pregnancy and 0.032 for smoking at delivery).

HES data were available for 667,432 deliveries from April 1, 2010 to March 31, 2011; of these, 5,655 were dropped as the mother’s age group, IMD and/or PCT of residence were missing, leaving 661,777 (99.2 %) deliveries in which to estimate smoking prevalence.

**Fig. 1:**
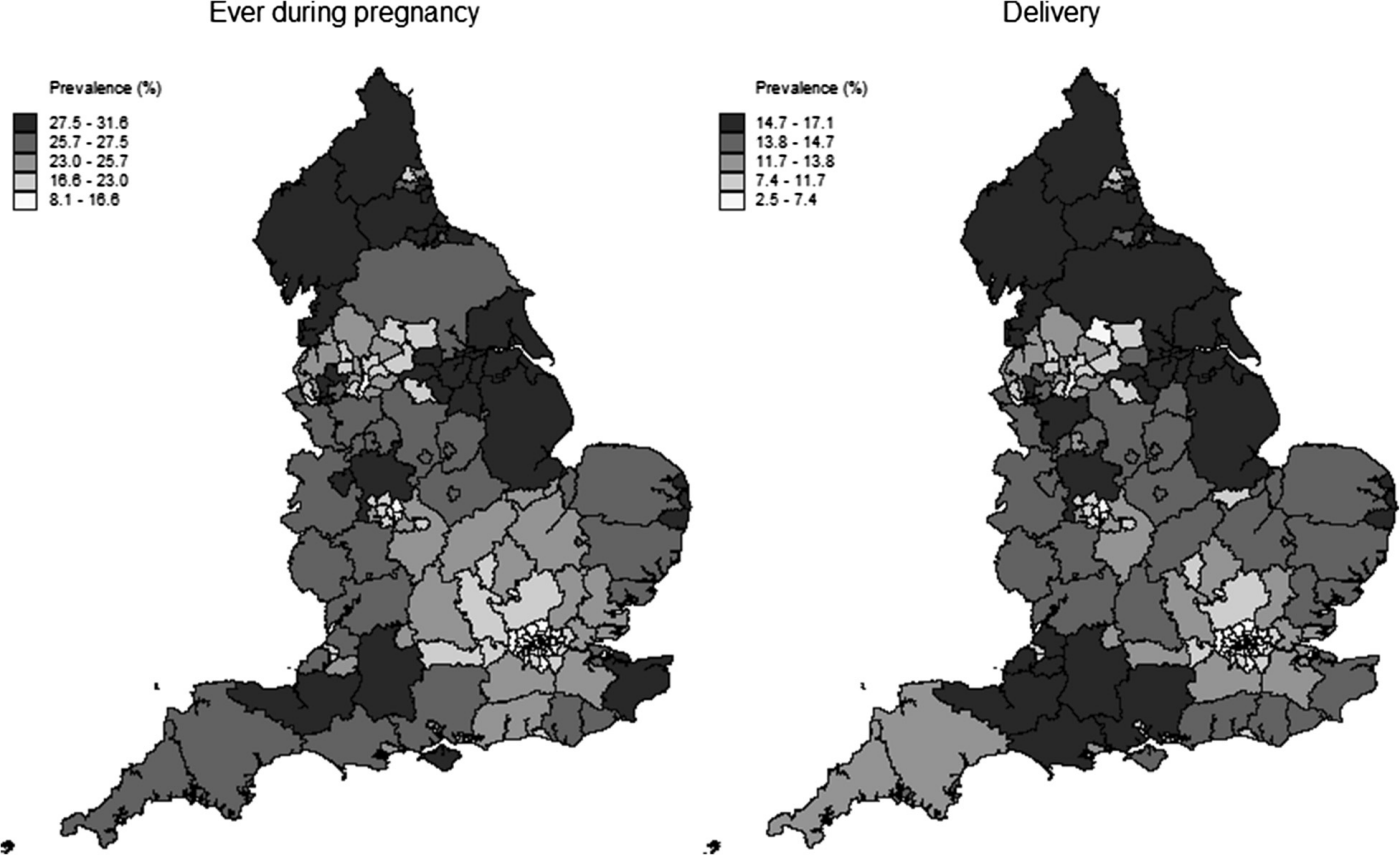
Synthetic estimates of smoking prevalence (by quintile) by PCT

Figure 1 shows the geographical variation in the synthetic estimates of the expected prevalence of smoking during pregnancy and at delivery by PCT. The figures used to produce these maps, plus the 95 % credible interval for each PCT, are presented in the supplementary material (Additional file 4). A color version of the map is available in the supplementary material (Additional file 5).

Broadly speaking, Fig. 1 shows a higher prevalence of smoking ever during pregnancy or at delivery in the more deprived areas where the population is predominantly of white or mixed ethnicity, such as the North West, North East, Lincolnshire, and east Kent. Lower expected figures are seen in areas such as the London boroughs and the West Midlands, which although relatively deprived in parts, have populations largely not of white or mixed ethnicity.

There was no evidence of substantial bias or heteroscedasticity in our synthetic estimates (see Additional file 6), though the very small number of women sampled in the IFS in some PCTs (16 PCTs had fewer than 20 survey respondents, and 5 had fewer than 10) makes comparisons between the IFS-based estimates and model-based synthetic estimates difficult.

**Fig. 2:**
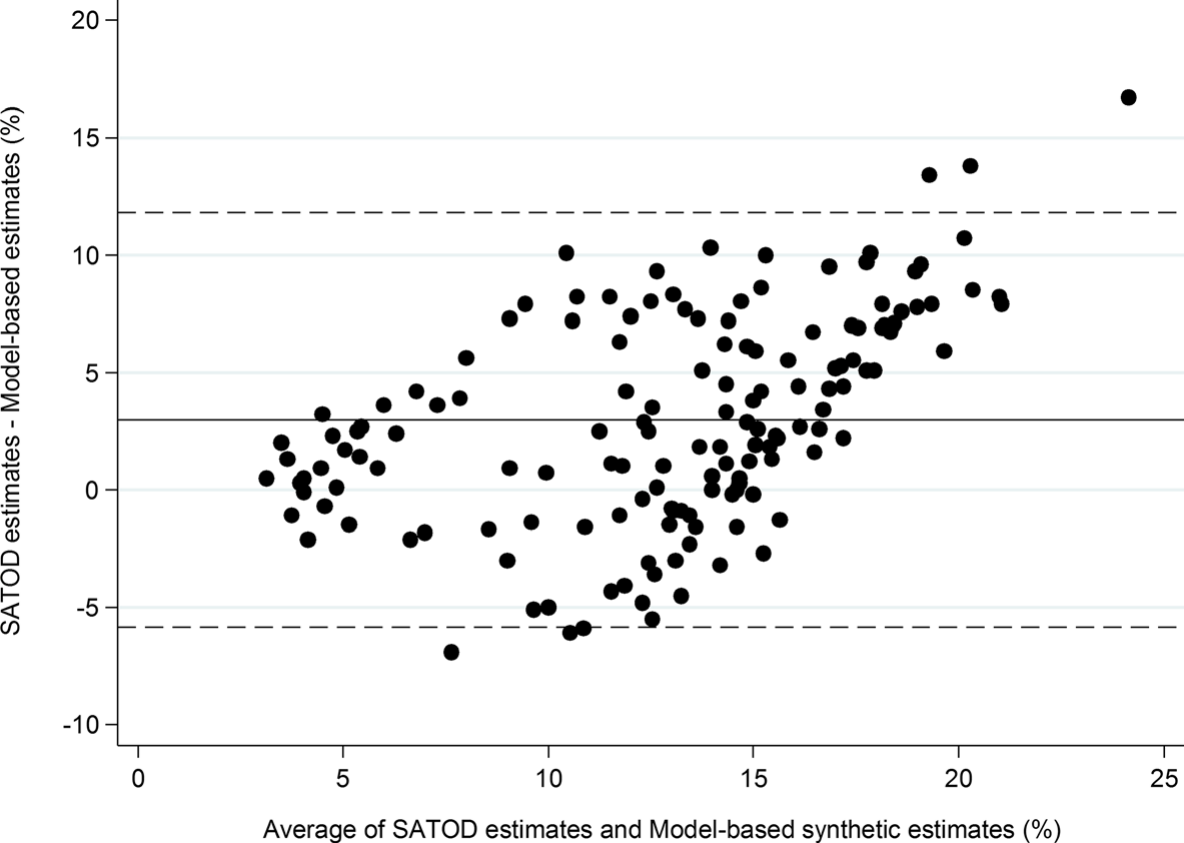
Bland-Altman plot to show agreement between model-based estimates of smoking at delivery and SATOD data (horizontal lines show the bias and 95 % limits of agreement)

The Bland-Altman plot in Fig. 2 illustrates the extent of agreement between SATOD data on the prevalence of smoking at delivery and our model-based synthetic estimates. The bias (the mean difference between the SATOD and model-based estimates for each PCT) is equal to 2.99 %, indicating that, on average, the SATOD estimates are higher than the model-based estimates. The limits of agreement are quite wide, showing there is large variability between the relative magnitudes of the two measures between PCTs. As the average of the SATOD and model-based estimates for each PCT increases, the difference between the two measures appears to increase (with SATOD data being the higher of the two).

## Discussion and conclusions

In this study we have demonstrated the potential to produce synthetic estimates for smoking prevalence during pregnancy at a geographical scale where data are not available routinely. Overall, these estimates appear to be valid.

These synthetic estimates represent a ”best guess” at the prevalence of smoking during pregnancy and at delivery based on the age group and deprivation level of women giving birth and the ethnic profile of the area in which they live. The figures are unlikely to be identical to data derived from survey-based methods. However, our figures for expected smoking prevalence at delivery show some agreement with SATOD data, though generally the synthetic estimates are slightly lower than SATOD figures. Based on the synthetic estimates, approximately 17 % of women in the worst-performing PCT were expected to be smoking at delivery. However, in the SATOD data, 28 PCTs had a smoking prevalence of at least 20 %. The SATOD estimate for smoking prevalence was outside the 95 % credible interval for a substantial number of PCTs (113 of 151, 74.8 %); PCTs with lower smoking prevalences tended to have 95 % credible intervals above the SATOD estimate and those with higher smoking prevalences tended to have 95 % credible intervals below the SATOD estimate.

Using SATOD data, Blackpool PCT has consistently been highlighted as having the highest figure for smoking prevalence at delivery in England—in 2010/11, 32.5 % of women were reported to be smoking [22]. However, our synthetic estimates suggest that the expected prevalence may have been much lower (15.8 %, 95 % CI 12.6–18.8), though the PCT still has one of the highest estimated smoking prevalences. An audit of data from Blackpool showed substantial misreporting of women’s smoking status [23]. Discrepancies were attributed to midwives using data recorded in first trimester booking records to impute smoking status at delivery even though some women would have quit [23]; midwives who smoked themselves not following recording guidelines [23] and finding discussing smoking difficult [4]; variations in service provider priorities [4]; as well as women themselves failing to accurately report their smoking status [4]. These findings may, at least in part, explain the discrepancy between SATOD data and our synthetic estimates. The national estimate of smoking prevalence at delivery is higher from SATOD data than from the IFS, with the difference being particularly marked in the North East and North West. This suggests that the discrepancies between our synthetic estimates and SATOD reflect differences in the data sources rather than issues with the synthetic estimation methodology. Crucially, however, our synthetic estimates represent an expected smoking prevalence and it remains possible that high-quality local survey or administrative data can demonstrate higher or lower actual levels of smoking.

Caution is needed, however, when interpreting our synthetic estimates and their credible intervals and we acknowledge there are some limitations in our methods. The IFS, used to predict the probability of smoking according to women’s characteristics, asks mothers retrospectively about their smoking during pregnancy and there is no biochemical validation of this measure. Data from women aged 20–24 in the United States demonstrated that 26.4 % of pregnant women identified as smokers based on serum cotinine levels did not disclose their smoking status in a face-to-face health survey interview [24]. Although the IFS is an anonymous online or postal questionnaire, there may still be some bias in the reporting of smoking status; women may give what they deem to be the socially-acceptable response, or they simply might not accurately remember the timing of changes in their smoking status during pregnancy.

Just three variables, age group, deprivation, and ethnicity, were included in the multilevel models used to derive the synthetic estimates of smoking prevalence. Small area estimates of smoking in the general population have considered other variables such as tenure, car ownership, unemployment, and overcrowding [8]. Data on these, plus other drivers of smoking behavior during pregnancy such as parity and partner smoking, may have helped to improve the synthetic estimates. However, such data were either not available, or not available in a comparable format, from both the IFS and HES datasets.

Our use of HES data which describe deliveries in NHS hospitals will have underestimated the number of pregnancies in an area in a year and thus the number of women who may have smoked. Approximately 3 % of all live births registered in England in 2010 occurred outside of NHS establishments, and this varied from 1.4 % in the north east to 4 % in the eastern region [25]. The incidence of non-hospital birth also increases with maternal age. For example, 1.3 % of deliveries where the mother is aged under 20 occurred outside of hospital in 2010, rising to 3.9 % in women age 30+. The prevalence of smoking reduces with increasing maternal age and therefore synthetic estimates based on HES data may have overestimated smoking prevalence, more so in regions where a higher proportion of births occur outside of NHS establishments. The use of HES data will also fail to capture pregnancies which end in miscarriage (approximately one in four pregnancies) though stillbirths are captured. For some of these women smoking would have contributed to their pregnancy loss [26], thus our methods will under-count an important group of women.

The PCTs used as the geographical area for synthetic estimation here were abolished in 2013 and replaced by 211 Clinical Commissioning Groups (CCGs) each serving an adult population of between 50,000 and 725,000 people [27]. The most recent IFS data available at the time of this study were from 2010 and thus we chose to use HES data from the 2010/11 financial year. It is possible that the relationship between women’s characteristics and their smoking behavior may change over time, between releases of the quinquennial IFS. However, with future releases of the IFS, and more recent HES data, the synthetic estimation process could be repeated to produce expected estimates of smoking prevalence by CCG, or a parameter for trend over time could be incorporated into the modeling process. Local-level data such as these might be useful to monitor the impact of smoking cessation interventions or services [7], for example the NHS Stop Smoking Services for Pregnancy.

On November 1, 2014, new information standards were introduced requiring all NHS-commissioned maternity services in England to routinely collect self-reported information on smoking status and daily cigarette consumption from pregnant women attending their booking appointment [5]. Data on women’s smoking status at the time of booking have been routinely collected in Scotland for several years as part of the Scottish Morbidity Record [28]. At present it is not possible to accurately ascertain smoking status at the time of booking from IFS data—the discrete response categories give women the option to report quitting on confirmation of pregnancy, which may have been several weeks before the booking appointment, or quitting, at an unspecified time, later in pregnancy. Synthetic estimation could, however, be used to derive local-level estimates for the expected number of smokers quitting on confirmation of pregnancy and later, to complement and help validate routinely-collected data on smoking at booking. The flexibility of the synthetic estimation method means estimates can be derived for bespoke geographical areas, or population sub-groups within areas, for which routinely-collected data may not be easily available. Such flexibility may be useful to enable monitoring of health-related inequalities between and within areas [7].

This approach to estimating the prevalence of smoking at specific time points or ever during pregnancy for small geographical areas is potentially quicker and cheaper than collecting survey data. If accepted as valid, these figures could be used to identify areas with high or unchanging smoking prevalence and improve the commissioning of targeted services to help women to quit.

## Additional files

Additional file 1:
**Overview of methods and calculation of Bayesian 95 % credible intervals.** (DOCX 46 kb) Additional file 2:
**Normal probability plots of PCT-level residuals from the IFS models.** (PDF 83 kb) Additional file 3:
**Geographical variation in PCT-level residuals from the IFS models.** (PDF 71 kb) Additional file 4:
**Point estimates and 95 % credible intervals for the prevalence of smoking ever during pregnancy and at delivery by PCT.** (XLSX 49 kb) Additional file 5:
**Synthetic estimates of smoking prevalence (by quintile) by PCT (color).** (PDF 67 kb) Additional file 6:
**Comparison of IFS-based estimates and model-based synthetic estimates.** (PDF 54 kb)

## Abbreviations

ESS: Estimated Sample Size
HES: Hospital Episode Statistics
IFS: Infant Feeding Survey
IGLS: Iterative Generalized Least Squares
IMD: Index of Multiple Deprivation
LSOA: Lower Super Output Area
MCMC: Markov Chain Monte Carlo
NHS: National Health Service
PCT: Primary Care Trust
SATOD: Smoking At Time of Delivery
SSSP: Stop Smoking Service for Pregnancy
