# Supplementary material for: Small area synthetic estimates of smoking prevalence during pregnancy in England
Source: Popul Health Metr. 2015 Dec 9;13:34. doi: 10.1186/s12963-015-0067-8 (PMC4674906; doi:10.1186/s12963-015-0067-8)
Supplement: Additional file 1: — Overview of methods and calculation of Bayesian 95 % credible intervals. (DOCX 46 kb) [file 12963_2015_67_MOESM1_ESM.docx]

Additional file 1: Overview of methods and calculation of Bayesian 95% credible intervals

Derive the logit for smoking during pregnancy and at delivery for each combination of age group and IMD quintile

From Census, identify the proportion of women not of white or mixed ethnicity by PCT

Match by PCT of residence with

individual-level data (from IFS) on smoking, age group and IMD quintile

Add the logit for the proportion of women not of white or mixed ethnicity to derive logits for each age/IMD group within each PCT

Untransform logit from mean of the posterior distribution to derive probability of smoking during pregnancy and at delivery for each age/IMD group within each PCT

(a)

Derive counts of the number of women who gave birth in each PCT in each combination of age/IMD group (from HES)

(b)

Divide number of smokers by number of

women who gave birth to derive

**smoking prevalence**

Sum counts for age/IMD groups to give

expected number of smokers by PCT

Multiply (a) by (b)

Repeat for 1 million simulations

Add a logit to account for random variation at the level of the PCT, drawn from a normal distribution with mean zero and standard deviation equal to the square root of the unexplained PCT-level variance from the simulation

Untransform total logit for each PCT for each simulation

(c)

For each simulation, derive count of

expected number of smokers in each PCT

Rank estimates and select the 2.5^th^ and 97.5^th^ percentile as the credible intervals for the

expected number of smokers

Multiply (c) by (b)

Divide by number of women who gave birth to derive **credible intervals** for smoking

prevalence

Derivation of smoking status variables

IFS participants were asked the following questions about their smoking behaviour:

- Have you ever smoked cigarettes? (yes/ no)
- Have you smoked at all the last two years? (yes/ no)
- Do you smoke cigarettes at all now? (yes/ no)
- Did you smoke cigarettes at all during pregnancy, after you found out you were pregnant? (yes/ no)
- When did you finally give up? (before you knew you were pregnant/ as soon as you found out you were pregnant/ later on during your pregnancy/ after the birth)
- Since you knew about your pregnancy, did you do any of the following? (stopped smoking temporarily/ used nicotine replacement therapy/ cut down on the number of cigarettes smoked each day/ increase the number of cigarettes smoked/ none of these)

The IFS data providers used participants’ responses to these questions to generate a derived smoking status variable categorised as follows:

1 Never smoked

2 Gave up over a year before pregnancy

3 Gave up less than a year before pregnancy

4 Gave up on confirmation of pregnancy

5 Gave up later in pregnancy, stayed quit

6 Gave up, but started again

7 Cut down

8 Did not cut down

Categories 4 to 8 inclusive were used to indicate smoking at any point during pregnancy, including between conception and confirmation of pregnancy. Categories 6 to 8 inclusive were used to indicate smoking at delivery.
