# Supplementary figures and images for: Small area synthetic estimates of smoking prevalence during pregnancy in England
Source: Popul Health Metr. 2015 Dec 9;13:34. doi: 10.1186/s12963-015-0067-8 (PMC4674906; doi:10.1186/s12963-015-0067-8)

Additional file 2: Normal probability plots of PCT-level residuals from the IFS models

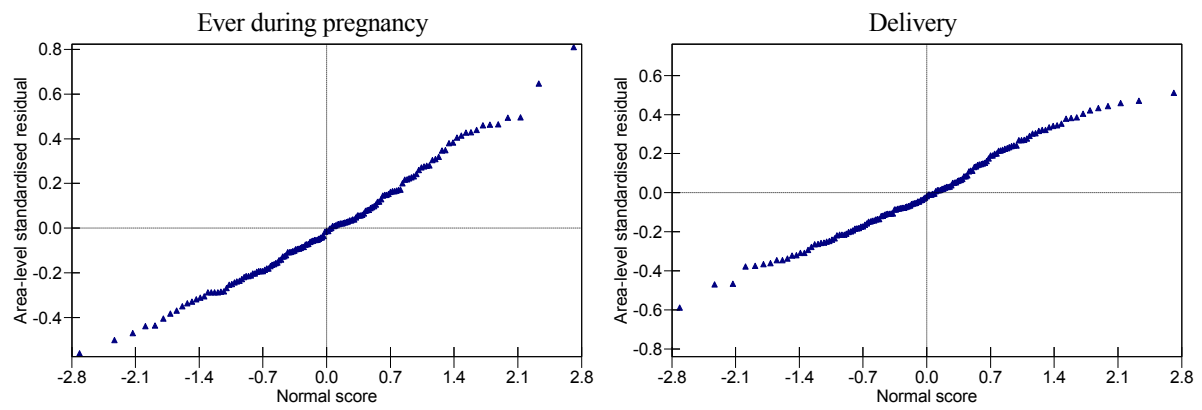

Supplement: Additional file 2: — Normal probability plots of PCT-level residuals from the IFS models. (PDF 83 kb) [file 12963_2015_67_MOESM2_ESM.pdf]

Additional file 3: Geographical variation in PCT-level residuals from the IFS models

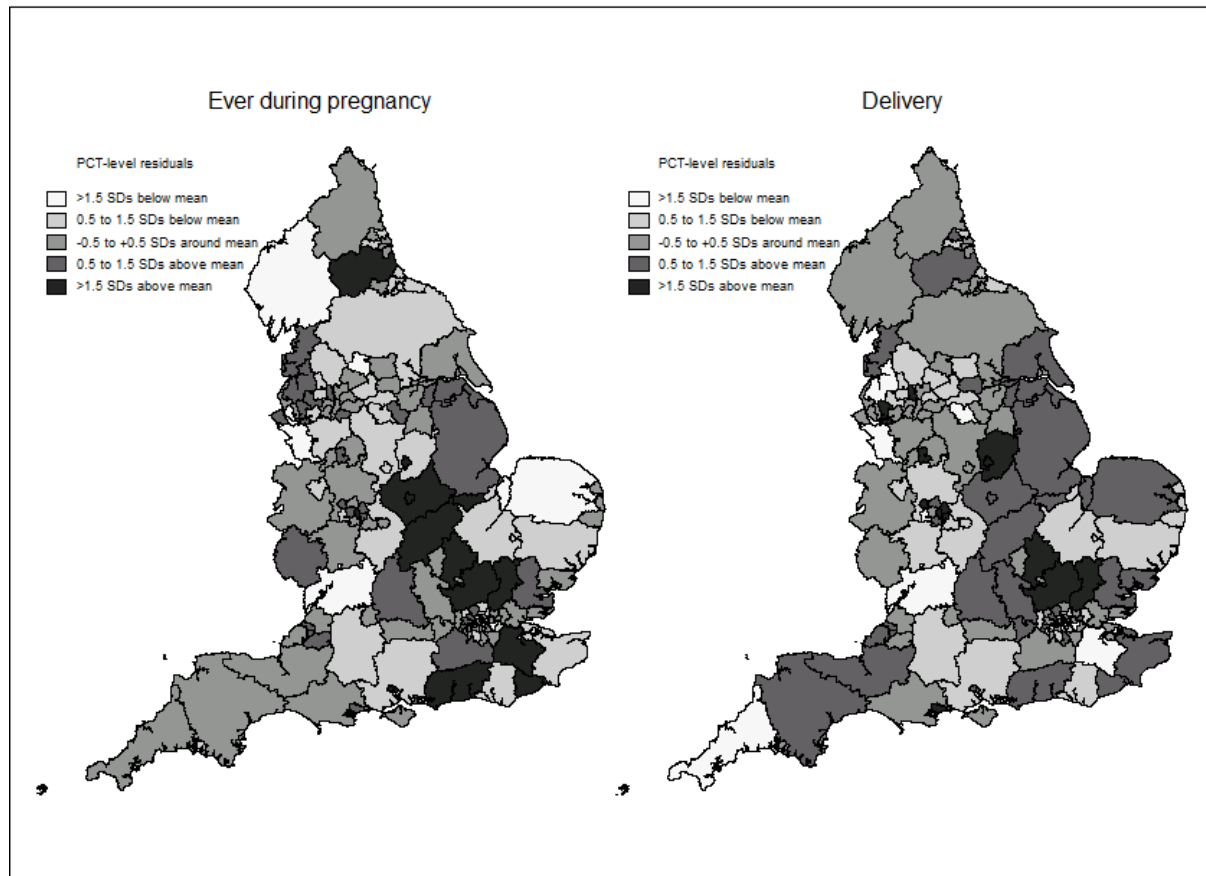

Supplement: Additional file 3: — Geographical variation in PCT-level residuals from the IFS models. (PDF 71 kb) [file 12963_2015_67_MOESM3_ESM.pdf]

Additional file 5: Synthetic estimates of smoking prevalence (by quintile) by PCT (colour)

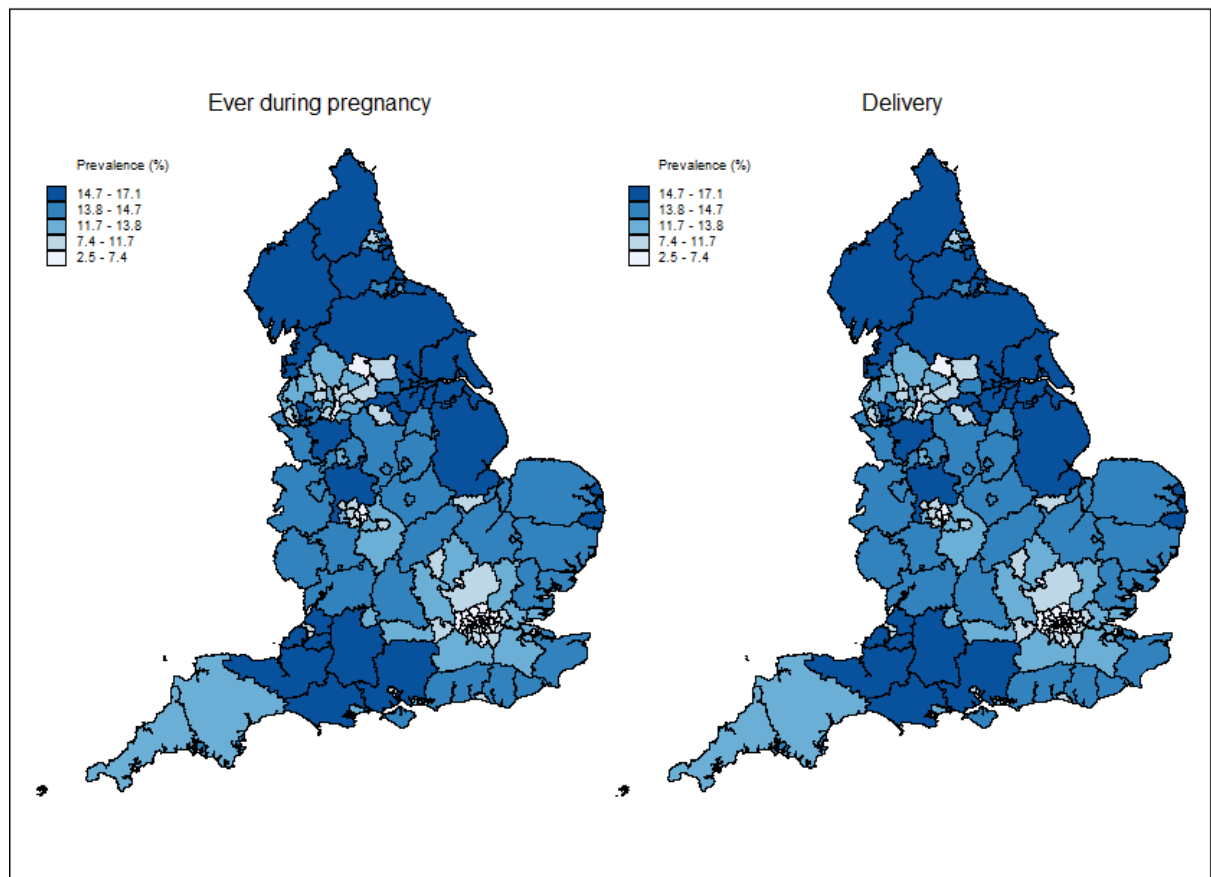

Supplement: Additional file 5: — Synthetic estimates of smoking prevalence (by quintile) by PCT (color). (PDF 67 kb) [file 12963_2015_67_MOESM5_ESM.pdf]

Additional file 6: Comparison of IFS-based estimates and model-based synthetic estimates

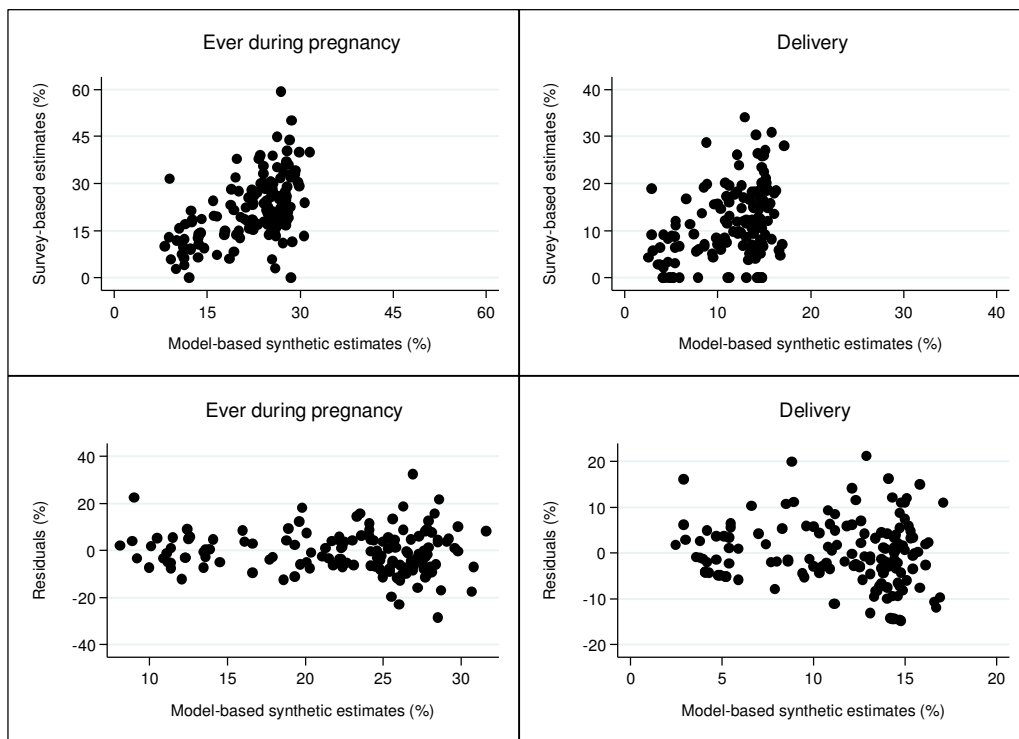

Supplement: Additional file 6: — Comparison of IFS-based estimates and model-based synthetic estimates. (PDF 54 kb) [file 12963_2015_67_MOESM6_ESM.pdf]
